# Supplementary material for: Dietary Intake of Polyphenols or Polyunsaturated Fatty Acids and Its Relationship with Metabolic and Inflammatory State in Patients with Type 2 Diabetes Mellitus
Source: Nutrients. 2022 Mar 4;14(5):1083. doi: 10.3390/nu14051083 (PMC8912460; doi:10.3390/nu14051083)
Supplement: Supplementary file 1 [file nutrients-14-01083-s001.zip › nutrients-1552901-supplementary.pdf]

Dietary intake of polyphenols or polyunsaturated fatty acids and its relationship with metabolic and inflammatory state in patients with type 2 diabetes mellitus.

Marcin Kosmalski [1], Anna Pękala-Wojciechowska [2], Agnieszka Sut [3], Tadeusz Pietras [4] and Boguslawa Luzak [5,*]

Table S1. The Bland-Altman analysis for total polyphenol and total fatty acid intake

	Polyphenols	Fatty acids	Desired value
Percent change [%]	2,69	1,79	<5

Percent change was estimated using Bland-Altman method.

Table S2. The analysis for different fatty acid compound intake estimated by FFQ1 and FFQ2

Fatty acids	Percent change [%]	Correlation coefficient (Rs)
SFA	2,15	0,99
MUFA	1,08	0,97
EPA+DHA	3,23	0,99
OMEGA-3	2,15	0,95
OMEGA-6	1,08	0,96
PUFA	1,08	0,93
Desired value	<5	>0,8

The percent change was determined with Bland-Altman method and correlation coefficient was determined by using Spearman rank correlation method.
SFA – saturated fatty acids, MUFA – monounsaturated fatty acids, PUFA – polyunsaturated fatty acids, EPA – eicosapentaenoic acid; DHA - docosahexaenoic acid

Table S3. The analysis for different phenolic compound intake estimated by FFQ1 and FFQ2

Polyphenols	Percent change [%]	Correlation coefficient (Rs)
Polyphenols	2,69	0,997
Flavonoids	4,30	0,993
Flawan-3-ols	2,15	0,987
Fenolic acids	2,15	0,958
Stilbens	2,15	0,870
Lignans	1,08	0,975
Desired value	<5	>0,8

The percent change was determined with Bland-Altman method and correlation coefficient was determined by using Spearman rank correlation method.

Table S4. Demographic and clinical characteristics of T2DM patients (n=129)

**Demographic variables**	**n (%)**
Gender:	
female	80 (62)
male	49 (38)
Educational level:	
low	45 (35)
medium	65 (50)
high	19 (15)
Physical activity level:	
low	80(62)
medium	38 (29)
high	11 (9)
Current smoking	19 (15)
Duration of diabetes*	3 (0; 27)
Following a diabetic diet	94 (73)
Familial diabetes	54 (42)
Diabetes complication:	
polyneuropathy	16 (12)
retinopathy	16 (12)
diabetic kidney disease	25 (19)
diabetic foot	4 (3)
Diabetes treatment:	
Untreated	12 (9)
MET monotherapy	51 (40)
PSU monotherapy	7 (5)
Pioglitazone monotherapy	1 (<1)
Insulin monotherapy	11 (8)
MET with SU	26 (20)
MET with SGLT2 inhibitors	4 (3)3
MET with insulin	12 (9)
MET with IDPP-4	1 (<1)
MET with IDPP-4 and SU	1 (<1)
MET with acarbose and insulin	1 (<1)
MET with SU and insulin	1 (<1)
SU with IDPP-4 and insulin	1 (<1)

*Data for Duration of diabetes are shown as a median (minimum; maximum). IDPP-4 – dipeptidyl peptidase-4 inhibitors, MET – metformin, SGLT2 - sodium-glucose cotransporter protein-2, SU – sulfonylureas.

Table S5. Descriptive characteristic of dietary intake of key food components

	**Mean**	**SD**	**Minimum**	**25th**	**Median**	**75th**	**Maximum**
Polyphenols [mg/day]							
total	1632	901	81	964	1656	2076	4723
flavonoids	810	478	8	408	793	1118	2392
flavan-3-ols	452	310	4	221	432	648	2161
phenolic acids	403	298	21	208	305	543	1655
stilbenes	0.67	1.15	0.00	0.03	0.19	0.74	6.63
lignans	34.6	67.9	0.8	9.0	18.0	36.7	559.2
Fas [g/day]							
SFA	29.4	20.1	1.5	18.7	26.1	35.7	179.8
MUFA	39.1	24.8	1.8	21.9	36.4	49.0	172.9
Total PUFA	19.4	14.9	1.8	9.4	16.8	26.0	122.8
PUFA omega 3	3.4	3.2	0.2	1.4	2.4	3.9	19.3
PUFA omega 6	15.1	12.0	1.5	7.2	12.8	19.6	101.5
EPA + DHA	0.65	0.96	0.00	0.19	0.33	0.69	6.77
Omega 6/3 proportion	5.6	2.9	0.7	3.4	5.3	6.9	18.8
Cholesterol [mg/day]	311	199	19	177	272	375	911

DHA - docosahexaenoic acid, EPA – eicosapentaenoic acid, Fas - fatty acids, MUFA – monounsaturated fatty acids, PUFA - polyunsaturated fatty acids, SFA – saturated fatty acids.

Table S6. Biochemical, haematological and anthropometric parameters (n=129)

	Mean	SD	Minimum	25th	Median	75th	Maximum
FG [mg/dl]	133	37	82	108	122	149	302
HbA_{1c} [%]	6.8	1.3	5.2	5.9	6.5	7.2	12.8
TCH [mg/dl]	182	51	98	149	180	209	332
LDL-CH [mg/dl]	106	42	22	76	101	128	253
HDL-CH cholesterol [mg/dl]	51	15	29	40	49	57	117
TG [mg/dl]	155	74	66	103	133	183	651
ALT [U/l]	28	17	11	19	25	33	163
AST [U/l]	26	10	10	19	23	31	64
Serum creatinine [mg/dl]	0.89	0.35	0.42	0.69	0.81	1.01	2.83
Leucocytes [$x10^3$/µl]	7.75	1.79	4.10	6.30	7.60	9.10	13.00
Lymphocytes [$x10^3$/µl]	2.18	0.71	0.70	1.80	2.10	2.60	4.79
Neutrophils [$x10^3$/µl]	4.70	1.46	2.10	3.60	4.60	5.64	9.00
Erythrocytes [$x10^6$/µl]	4.74	0.51	3.22	4.43	4.76	5.08	5.83
Haemoglobin [g/l]	14.2	1.4	9.6	13.4	14.4	15.0	17.3
Haematocrit [%]	42.2	3.9	30.2	40.2	42.7	44.7	50.7
Platelet count [$x10^3$/µl]	242	69	43	200	234	280	571
Mean platelet volume [fl]	10.5	1.0	8.3	9.7	10.4	11.2	13.1
PLR	122	54	43	85	112	141	389
NLR	2.41	1.21	0.44	1.58	2.21	2.89	8.56
MPVLR	5.40	2.09	2.42	4.03	4.86	6.11	13.57
SBP [mmHg]	140	17	100	129	140	151	185
DBP [mmHg]	80	10	60	74	80	85	110
BMI	33.1	7.7	18.2	28.2	32.4	36.4	60.5
Waist circumference	110	16	74	102	110	118	168
WHR	0.966	0.077	0.775	0.921	0.966	1.018	1.137

ALT - alanine aminotransferase activity, AST - aspartate aminotransferase activity, BMI – body mass index, DBP- diastolic blood pressure, FG- fasting glucose concentration, HbA1c – glycated haemoglobin, HDL-CH – HDL-cholesterol, LDL-CH – LDL-cholesterol, MPVLR - mean platelet volume-to-lymphocyte ratio, NLR - neutrophil-to-lymphocyte ratio, PLR - platelet-to-lymphocyte ratio; SBP – systolic blood pressure, TCH - total cholesterol, TG - triglycerides WHR – waist-to-hip ratio.
